# Soluble Free, Esterified and Insoluble-Bound Phenolic Antioxidants from Chickpeas Prevent Cytotoxicity in Human Hepatoma HuH-7 Cells Induced by Peroxyl Radicals

**DOI:** 10.3390/antiox11061139

**Published:** 2022-06-10

**Authors:** Adriano Costa de Camargo, Alina Concepción Alvarez, María Fernanda Arias-Santé, Juan Esteban Oyarzún, Marcelo E. Andia, Sergio Uribe, Paula Núñez Pizarro, Simón M. Bustos, Andrés R. Schwember, Fereidoon Shahidi, Raquel Bridi

**Affiliations:** 1 Nutrition and Food Technology Institute, University of Chile, Santiago 7830490, Chile; a24ca90@gmail.com (A.C.A.); ma.fernanda.arias@inta.uchile.cl (M.F.A.-S.); 2 Biomedical Imaging Center, School of Medicine, Pontificia Universidad Católica de Chile, ANID-Millennium Institute for Intelligent Healthcare Engineering—iHEALTH, Santiago 7820436, Chile; jeoyarzu@uc.cl (J.E.O.); meandia@uc.cl (M.E.A.); suribe@uc.cl (S.U.); 3 Departamento de Ciencias Vegetales, Facultad de Agronomía e Ingeniería Forestal, Pontificia Universidad Católica de Chile, Santiago 7820436, Chile; pjnunez@uc.cl (P.N.P.); smbustos@uc.cl (S.M.B.); aschwember@uc.cl (A.R.S.); 4 Department of Biochemistry, Memorial University of Newfoundland, St. John’s, NL A1C 5S7, Canada; fshahidi@mun.ca; 5 Departamento de Química Farmacológica y Toxicológica, Facultad de Ciencias Químicas y Farmacéuticas, Universidad de Chile, Santiago 8380000, Chile

**Keywords:** *Cicer arietinum* L., phenolic acids, flavonoids, reducing power, antiradical activity, hepatoprotection

## Abstract

Chickpeas are rich sources of bioactive compounds such as phenolic acids, flavonoids, and isoflavonoids. However, the contribution of insoluble-bound phenolics to their antioxidant properties remains unclear. Four varieties of chickpeas were evaluated for the presence of soluble (free and esterified) and insoluble-bound phenolics as well as their antiradical activity, reducing power and inhibition of peroxyl-induced cytotoxicity in human HuH-7 cells. In general, the insoluble-bound fraction showed a higher total phenolic content. Phenolic acids, flavonoids, and isoflavonoids were identified and quantified by UPLC-MS/MS. Taxifolin was identified for the first time in chickpeas. However, *m*-hydroxybenzoic acid, taxifolin, and biochanin A were the main phenolics found. Biochanin A was mostly found in the free fraction, while *m*-hydroxybenzoic acid was present mainly in the insoluble-bound form. The insoluble-bound fraction made a significant contribution to the reducing power and antiradical activity towards peroxyl radical. Furthermore, all extracts decreased the oxidative damage of human HuH-7 cells induced by peroxyl radicals, thus indicating their hepatoprotective potential. This study demonstrates that the antioxidant properties and bioactive potential of insoluble-bound phenolics of chickpeas should not be neglected.

## 1. Introduction

Consumption of chickpeas has been associated with different benefits to humans such as weight management, gut health, and improvements in cardiovascular diseases [1]. India is the main producer of chickpeas [2]. After a long period of decrease [3], consumption of this legume has increased in Chile during COVID-19 confinement, despite a decrease in its production in the country [2,4]. Beyond serving as a rich source of protein, fiber, minerals and vitamins, increasing evidence suggests that chickpeas are important sources of phenolic compounds. These metabolites play important roles associated with growth regulation as well as protection from sunlight and predators. Moreover, their antioxidant activity has been positively linked with improvements in some diseases (cardiovascular, cancer, arthritis) [5,6,7], and physiological benefits [8].

Identification of phenolic compounds in all their possible forms is important for optimal and real interpretation of their benefits. Phenolic compounds are divided into soluble or bound form. As soluble conjugates, phenolic compounds can be bound covalently to different metabolites such as fatty acids (soluble esters) or insoluble macromolecules such as proteins, cellulose, and arabinoxylans (insoluble-bound phenolics, IBPs) [9]. Some studies have identified or quantified different groups of phenolic compounds in legume seeds, including chickpeas [9,10,11].

There is much information about the soluble phenolic compounds that are frequently analyzed upon obtaining a crude extract without any further fractionation and/or hydrolysis. Nevertheless, the interactions with intestinal microorganisms and analytical methods for satisfactory extraction of IBPs have not been properly addressed because insoluble-bound phenolics are often ignored [12]. Some free and esterified phenolic compounds have been identified in cereal grains and legume seeds, including chickpeas [11,13,14]. Only one study has detected phenolic compounds in three forms in one chickpea variety and other beans [15].

As for chickpeas, only two phenolic acids and three flavonoids have been identified in the free fraction, whereas the fractions released from the esterified (soluble conjugate) and insoluble-bound forms showed the presence of two phenolic acids [16]. Therefore, a clear gap exists in the literature, especially about insoluble-bound phenolic acids, flavonoids, and isoflavones. Soybeans and products thereof are important sources of isoflavones [17,18,19]. Recently, chickpeas have also been shown to have isoflavones as their main phenolics [20] and hence may be regarded as a possible soybean substitutes and a source of isoflavones.

In previous studies, we compared three of these varieties according to their affected flavonoid profiles due to climate changes in two years [21]. We found differences in free and esterified flavonoids detection in ‘Local Navidad’, ‘California-INIA’, and ‘Alfa-INIA’, but the fraction containing insoluble-bound compounds was not considered. Currently, no single study exists on the characterization and quantification of different phenolic compound fractions in these Chilean chickpea varieties. Therefore, our results are of both local and international interest.

Studies have shown that IBPs have similar effects to prebiotics [11]. This phenolic fraction could lead to the development of bioactive functions in different cells of the organism. However, their benefits have rarely been studied because they depend on factors such as synergic effects, uptake, absorption, type, quantity, and release for microorganisms, among others [10]. Therefore, accurate the characterization and functions of all phenolic varieties in the three aforementioned fractions are important as a first step to anticipating their real bioavailability, bioaccessibility, and metabolism.

The available information on IBPs are insufficient for their inclusion in phenolic databases such as those created by the United States Department of Agriculture (USDA). This limited or lack of information may lead to an inappropriate estimation of dietary intake [22] and jeopardize decision making with respect to the effects of food processing that may affect IBPs such as non-ionizing and ionizing radiation, enzyme treatment, fermentation, and germination, among others. To fill the existing gap, phenolic compounds in the free, esterified, and insoluble-bound fraction of Chilean chickpea varieties (‘Local Santo Domingo’, ‘Local Navidad’, ‘California-INIA’, and ‘Alfa-INIA’) were characterized for their total phenolic content (TPC), phenolic acids, flavonoids, and isoflavonoids by UPLC-MS/MS, as well as their reducing power, and antiradical activity. Moreover, we evaluated the hepatoprotective potential of free, soluble esters and IPBs against oxidative damage induced by peroxyl radicals using human hepatoma HuH-7 cells.

## 2. Materials and Methods

### 2.1. Plant Material and Reagents

Four chickpea kabuli-type varieties were evaluated, two cultivars were from INIA (Chile’s Agricultural Research Institute): ‘Alfa-INIA’ and ‘California-INIA’, and two landraces: ‘Local Navidad’ and ‘Local Santo Domingo’, currently uncharacterized, but used by local farmers from Navidad (O’Higgins region) and ‘Local Santo Domingo’ (Valparaiso region), respectively. Sodium hydroxide (NaOH), 2,4,6-tri(2-pyridyl)-S-triazine (TPTZ), hydrochloric acid (HCl), water (H_2_O), methanol (MeOH), diethyl ether, ethyl acetate, acetone, Folin–Ciocalteu reagent, sodium carbonate (Na_2_CO_3_), gallic acid, ferric chloride, acetate buffer, ethanol, Trolox, 2,2′-azo-bis(2-amidinopropane) dihydrochloride (AAPH), phenolic acids (*m*-hydroxybenzoic, cinnamic, gallic, *p*-coumaric, ferulic, syringic, and sinapic acid), flavonoids (luteolin, kaempferol, taxifolin, isorhamnetin, and rutin), and isoflavonoids (daidzein, formononetin, genistein, and biochanin A) were purchased from Sigma-Aldrich (St. Louis, MO, USA) or Merck (Darmstad, Germany). Fetal bovine serum (FBS), antibiotic and antimycotic solution, Triton X-100 were bought from Sigma-Aldrich (St. Louis, MO, USA).

### 2.2. Extraction of Free and Esterified Phenolics

Free and esterified phenolic compounds were extracted from dry chickpea samples as reported previously [21,23]. These samples were divided into portions and mixed with water (1:3, *w*/*v*). Then, chickpeas were macerated at 5 °C for 15 h. Once the water was drained, samples were added to a solution of methanol/acetone/water (7:7:6, *v*/*v*/*v*) and homogenized for 2 min using a blender (Oster, Model BRLY07-Z00, Milwaukee, WI, USA). The samples were centrifuged for 5 min at 4000× *g* (Z-326 K, Hermle Labortechnik GmbH, Wehingen, Germany). The supernatant was transferred to another container tube and this extraction was replicated two more times. The organic solvent was then removed using a rotaevapor at 40 °C. The residual aqueous solution was mixed with HCl (6 M) to reach pH = 2. Afterward, to this solution was added diethyl ether/ethyl acetate (1:1, *v*/*v*) for the extraction of free phenolic compounds. This extraction cycle was repeated five times and the organic solution was transferred to a glass container for evaporation. The samples were then dried under vacuum at 40 °C and the water phase was mixed with NaOH (4 M; 1:1, *v*/*v*). Esterified phenolic compounds were released upon hydrolysis at 23–25 °C for 4 h. Afterward, HCl (6 M) was added to the samples for acidification to reach pH = 2 and the liberated compounds (originally esterified) were collected as free phenolics. Finally, both fractions (free and esterified) were separately reconstituted with MeOH (HPLC grade) and the samples were stored in a refrigerator at −80 °C until analysis.

### 2.3. Insoluble-Bound Phenolic Compounds Extraction

IPBs were extracted from the remaining residues after extraction of soluble phenolics. This method was described by de Rezende et al. [22,23]. First, the sample was mixed with NaOH (4 M, 20 mL for each g of the sample) and incubated at 23–25 °C for 4 h. Then, to the blend was added HCl (6 M) until pH = 2 and each was extracted with diethyl ether/ethyl acetate (1:1, *v*/*v*) five times. Subsequently, the solvent was evaporated under vacuum at 40 °C, followed by freeze drying of the sample. Finally, the extract was reconstituted with MeOH (HPLC grade) for analysis.

### 2.4. Total Phenolic Content

Total phenolic content was determined according to Singleton et al. [24] considering the modifications described by Bridi et al. [25]. Folin–Ciocalteu reagent (125 μL) was mixed with a diluted solution of samples (25 μL) and Na_2_CO_3_ at 7.5% (100 μL). This homogenized preparation was added into each cell of polystyrene microplates in Cytation 5 multimode microplate reader from BioTek Instruments, Inc. (Winooski, VT, USA). The samples were then incubated at 37 °C for 30 min. The absorption was read at 756 nm in the microplate reader. Total phenolic determination was performed by using a gallic acid calibration curve (10 to 180 mg/L). The results were expressed as milligrams of gallic acid equivalents (GAE) per 100 g of sample (mg GAE/100 g). The results are reported as means with standard deviations (SD) of 3 independent determinations.

### 2.5. Ferric Reducing Antioxidant Power

The ferric reducing antioxidant power determination was carried out according to Bridi et al. [25]. First, 10 parts of acetate buffer (0.3 M, pH 3.6), 1 part of TPTZ (10 mM), and 1 part of ferric chloride (20 mM) were mixed. This solution (270 μL) was homogenized with 30 μL of the diluted sample and incubated at 37 °C for 30 min. The absorbance was then read at 594 nm using a Cytation 5 multimode microplate reader from BioTek Instruments, Inc (Winooski, VT, USA). Trolox (5−30 μM) were used as positive controls. The obtained values were expressed as μmol Trolox equivalents per g of sample (μmol TE/100 g) and are reported as means with standard deviations (SD) of 3 independent determinations.

### 2.6. Oxygen Radical Absorbance Capacity

The oxygen radical absorbance capacity (ORAC) was determined according to Bridi et al. [25] using a fluorescent microplate reader (Cytation 5 from BioTek Instruments Inc). The wavelengths used were 493 nm (excitation) and 515 nm (emission). The intensity decline in samples allowed evaluation of fluorescein consumption. Trolox (2−10 μM) was used as a standard, while AAPH was used as peroxyl ion generator. The obtained values were expressed as μmol Trolox equivalents per 100 g of sample (μmol TE/100 g) and reported as means with standard deviations (SD) of 3 independent determinations.

### 2.7. UPLC-MS/MS Analysis

Free, esterified, and insoluble-bound phenolics were detected/studied through an ABSciex triple Quad 4500 mass spectrometer supplied with an electrospray (TurboV) interface combined to an Eksigent Ekspert Ultra LC100 with an Ekspert Ultra LC100-XL autosampler system (AB/Sciex, Concord, ON, Canada). Electrospray in the negative mode was employed and the following parameters were employed: curtain gas (CUR) = 30; collision gas (CAD) = 10; ion spray voltage (IS) = −4500; temperature (TEM) = 650; ion source gas 1 (GS1) = 50; ion source gas 2 (GS2) = 50; entrance potential (EP) = 10. Chromatographic separation was carried out by employing a gradient elution with (A) 0.1% formic acid and (B) methanol as the mobile phase, using the following protocol: 0–1 min, 5% B; 1–12 min, 5–50% B; 12–13 min 50–50% B; 13–14 min, 50–5% B; and 14–15 min, 5% B. The apparatus was handled utilizing an injection volume of 10 μL, a flow rate of 0.5 mL/min, and an end-capped column (LiChrospher 100 RP-18; 125 mm × 4 mm i.d., 5 μm; Merck, Darmstadt, Germany) kept at 50 °C. Since a higher temperature has been found to improve chromatographic separation, using the same column, other authors employed temperatures ranging from 45–50 °C to identify and quantify different compounds, including phenolic acids and flavonoids [26,27,28,29,30]. Calibration curves for quantification were built utilizing commercially available standards. Limits of detection (LOD), limit of quantification (LOQ) and r^2^ of the plotted graphs were: gallic acid (LOD = 41 ppb, LOQ = 124 ppb, and r^2^ = 0.9988); *p*-coumaric acid (LOD = 124 ppb, LOQ = 377 pbb, and r^2^ = 0.9911); ferulic acid (LOD = 110 ppb, LOQ = 334 ppb, and r^2^ = 0.9944); syringic acid (LOD = 55 ppb, LOQ = 167 ppb, r^2^ = 0.9995); sinapic acid (LOD = 120 ppb, LOQ = 364 ppb, and r^2^ = 0.9984); daidzein (LOD = 108 ppb, LOQ = 328 ppb, and r^2^ = 0.9906); genistein (LOD = 105 ppb, LOQ = 319 ppb, and r^2^ = 0.9908); luteolin (LOD = 136 ppb, LOQ = 412 ppb, and r^2^ = 0.9965); kaempferol (LOD = 390 ppb, LOQ = 1181 ppb, and r^2^ = 0.9905); isorhamnetin (LOD = 198 ppb, LOQ = 599 ppb, r^2^ = 0.9963); and rutin (LOD = 249 ppb, LOQ = 756 ppb, and r^2^ = 0.9939). Table 1 shows the parameters used for compound identification.

### 2.8. Cytotoxicity and Hepatoprotective Activity

Human hepatoma HUH-7cells supplied by ATCC (American Type Culture Collection) were used. These cells were grown in 75 cm^2^ flasks using DMEM with high glucose content, supplemented with 10% FBS and 1% antibiotic and antimycotic solution. The cells were kept in a humidified atmosphere with 5% CO_2_–95% air at 37 °C. HUH-7 cells were seeded at a density of 50,000 cells per well in 96-well plates. After 24 h, cells were incubated with phenolic extracts of chickpea (1–10) at different dilutions (1/10, 1/100, 1/1000, and 1/10,000). The induction of cell damage was carried out using 2,2′-azobis (2-amidinopropane) dihydrochloride (AAPH) for 24 h at different concentrations (0.002–200 mM). Triton X-100 at 1% for 10 min was applied as a positive control of cell death. Cell mortality was determined by reduction of resazurin (Alamar Blue Assay) and measuring fluorescence (560 nm excitation/590 nm emission) using a Cytation™ 5 multi-mode microplate reader from BioTek Instruments, Inc. (Winooski, VT, USA) [31,32,33]. The results are expressed as a percentage of the control conditions of three independent experiments and three replicates per experiment.

### 2.9. Statistical Analysis

The results of the hepatoprotective activity and cytotoxicity tests are presented as means ± standard deviation (SD). Statistical comparisons between two groups were evaluated with Mann–Whitney statistical test. Statistical comparison between 3 or more groups was performed with one-way ANOVA followed by Tukey post-hoc test. Significance was accepted at *p* < 0.05.

## 3. Results

### 3.1. Total Phenolic Content (TPC) and Ferric Reducing Antioxidant Power (FRAP)

The TPC showed different levels of contributions of soluble (free and esterified) and insoluble-bound compounds in the four varieties of chickpeas studied (Table 2). Considering all fractions (free + esterified + insoluble-bound forms), ‘Local Santo Domingo’ had the highest TPC (31.5 mg GAE/100 g) followed by ‘California-INIA’ (25.1 mg GAE/100 g), ‘Local Navidad’ (22.9 mg GAE/100 g), and ‘Alfa-INIA’ (17.3 mg GAE/100 g). The main contribution to this total was from IBPs in most varieties (53% for ‘California-INIA’, ‘Local Navidad’, and ‘Local Santo Domingo’), except in ‘Alfa-INIA’, where a greater contribution of the free phenolic compounds fraction was noted (51%). The TPC in these fractions was significantly different (*p* < 0.05).

The FRAP was evaluated in all varieties of chickpeas (Table 2). The ability of the phenolic compounds to reduce Fe^3+^ to Fe^2+^ was higher in ‘Local Santo Domingo’ (70.4 μmol TE/100 g), followed by ‘Alfa-INIA’ (52.7 μmol TE/100 g), ‘California-INIA’ (44.1 μmol TE/100 g), and ‘Local Navidad’ (33.4 μmol TE/100 g). In three of the evaluated varieties, the fraction of phenolic compounds with the highest levels of FRAP was IBPs while free and esterified fractions made the lowest contribution. Nevertheless, ‘Local Navidad’ was the only variety with a lower value in the IBPs fraction. However, the insoluble-bound fraction contributed 40% of the reducing power of ‘Local Navidad’, which is not negligible. In general, the FRAP among the tested fractions was significantly different (*p* < 0.05) and the contribution of IBPs ranged from 35 to 44%.

The oxygen radical absorbance capacity (ORAC) showed different values in all chickpea varieties (Table 2). ‘California-INIA’ had the highest antioxidant capacity (2049.3 μmol TE/100 g) of all evaluated varieties, while ‘Alfa-INIA’ had the lowest value (706.5 μmol TE/100 g). In all chickpea samples, the major contribution to antioxidant capacity was from the insoluble-bound compounds. Moreover, the lower values were due to the esterified fraction compared with the free fraction in all varieties studied.

### 3.2. Identification of Phenolics Compounds by LC-MS/MS

The identification of phenolic compounds in all chickpea varieties was carried out using multiple reaction monitoring (MRM) by LC-MS/MS (Table 1). The different parameters of analysis allowed identification of phenolic acids, flavonoids, and isoflavonoids in the soluble and IBPs fractions of the four chickpea varieties evaluated.

We identified phenolic acids (n = 6), flavonoids (n = 5), and isoflavonoids (n = 4) in at least one of the three fractions in each chickpea variety (Table 3). The phenolic acids observed in these chickpeas were m-hydroxybenzoic acid, *p*-coumaric acid, cinnamic acid, ferulic acid, syringic acid, and sinapic acid. The compounds m-hydroxybenzoic acid and *p*-coumaric acid were detected in the soluble (free and esterified) and IBP fractions in the four varieties. Moreover, other phenolic acids such as cinnamic (present only in the free form), ferulic (always present as soluble esterified and insoluble-bound), syringic (always present as soluble esterified and insoluble-bound), and sinapic (present only in the free form) acids were not found in all fractions. The flavonoids identified were luteolin, kaempferol, taxifolin, isorhamnetin and rutin; among them, only kaempferol, rutin, and isorhamnetin were detected in all fractions of the four chickpea varieties, while the others were present in one or two fractions. Besides kaempferol and rutin, taxifolin was also present in the insoluble-bound phenolic fraction. Furthermore, we observed biochanin A as the predominant isoflavonoid in all fractions. In addition, the others were present in one or two forms (daidzein in the free form or formononetin in free and IBPs; genistein in esterified and IPBs fractions were present in chickpea varieties).

The identified compounds were quantified in the different fractions (Table 3). Six compounds (luteolin, kaempferol, isorhamnetin, rutin, daidzein, and genistein) were detected only in trace amounts. The total quantified phenolic acids were present at 1.6–458.0 µg/100 g of sample. Among these, m-hydroxybenzoic acid was the unique compound with high levels (217.2–458.0 µg/100 g of sample) in the four varieties, while cinnamic acid was present at 1.6–2.8 µg/100 g of sample. This last compound was quantified only in the free fractions of different varieties, except in ‘Local Santo Domingo’, where only trace amounts were present. Taxifolin was the only flavonoid compound with a quantifiable level in all fractions (9.4–155.5 µg/100 g of sample), while formononetin (an isoflavonoid) was only quantifiable in the free fraction. Furthermore, we detected high levels of biochanin A (6296.1–8380.0 µg/100 g of sample). This compound was the most predominant in the tree fractions in all varieties.

### 3.3. Cytotoxicity and Hepatoprotective Potential

**Figure 1:**
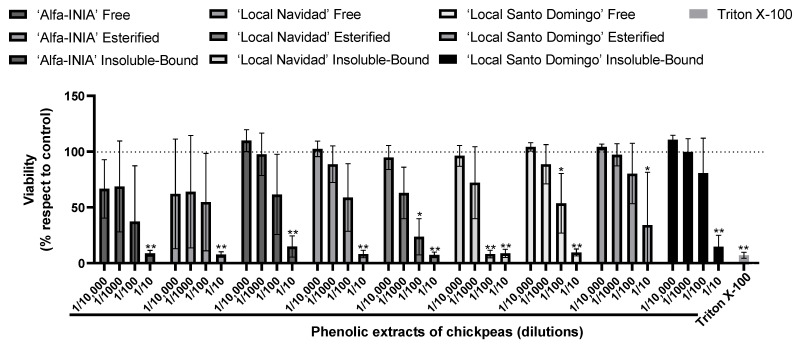
Maximum concentration of phenolic extracts of chickpea. Cell viability evaluated by Alamar blue of HUH-7 cells treated or 24 h with phenolic extracts of chickpeas at different dilutions (1/10, 1/100, 1/1000 and 1/10,000). Positive control of cell death, cells treated with Triton X-100 at 1% for 10 min. Data are expressed as percentage of viability with respect to the control cells. Data are shown as mean ± SD (n = 3). A one-way ANOVA statistical test was performed followed by Tukey test. Statistically significant differences compared to the control group (cells without treatment) (* *p* < 0.05, ** *p* < 0.01).

**Figure 2:**
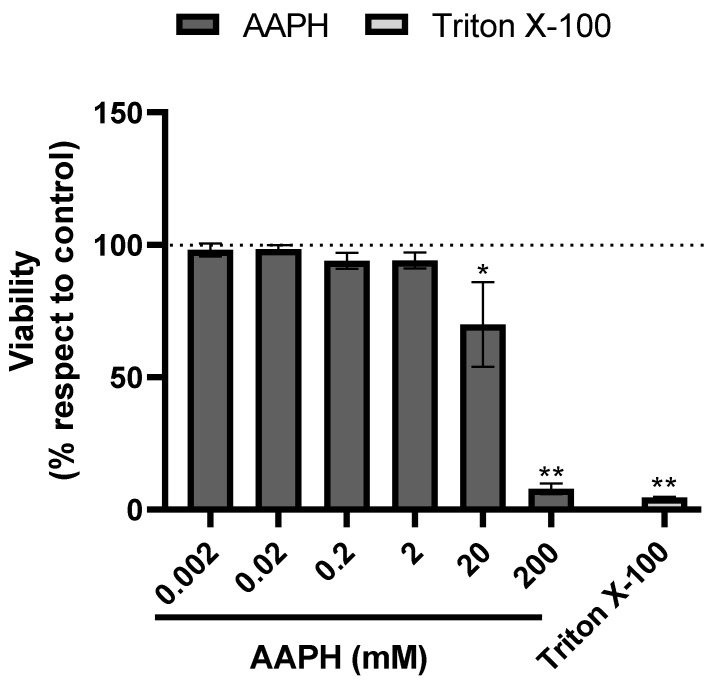
AAPH-induced cell death. Cell viability evaluated by Alamar blue of HUH-7 cells treated with 2,2′-Azobis (2-amidinopropane) dihydrochloride (AAPH) for 24 h at different concentrations (0.002–200 mM). Positive control of cell death, cells treated with Triton X-100 at 1% for 10 min. Data are expressed as percentage of viability with respect to the control cells only with vehicle. Data are shown as mean ± SD (n = 3). A one-way ANOVA statistical test was performed followed by Tukey test. Statistically significant differences compared to the control group (cells without treatment) (* *p* < 0.05, ** *p* < 0.01).

First, HUH-7 cells were incubated for 24 h using four different dilutions (1/10 to 1/10,000) of phenolic extracts of chickpeas. The cell viability was calculated compared to the control cells (untreated cultures), which were considered to present 100% cell viability. To evaluate the cytotoxicity of the samples on HUH-7 cells, we used the Alamar blue viability assay. In general, apart from the highest concentration of phenolic extract of chickpeas (1/10), all other concentrations were noncytotoxic (Figure 1). The hepatoprotective activity of phenolic extract of chickpeas was determined under equivalent conditions to those used in the experiments carried out to evaluate cytotoxicity. The oxidative insult to HUH-7 cells was induced by the potent oxidant AAPH, at concentrations from 20 μM to 200 mM generate cell death, for hepatoprotection experiments we used 200 μM of AAPH (Figure 2).

**Figure 3:**
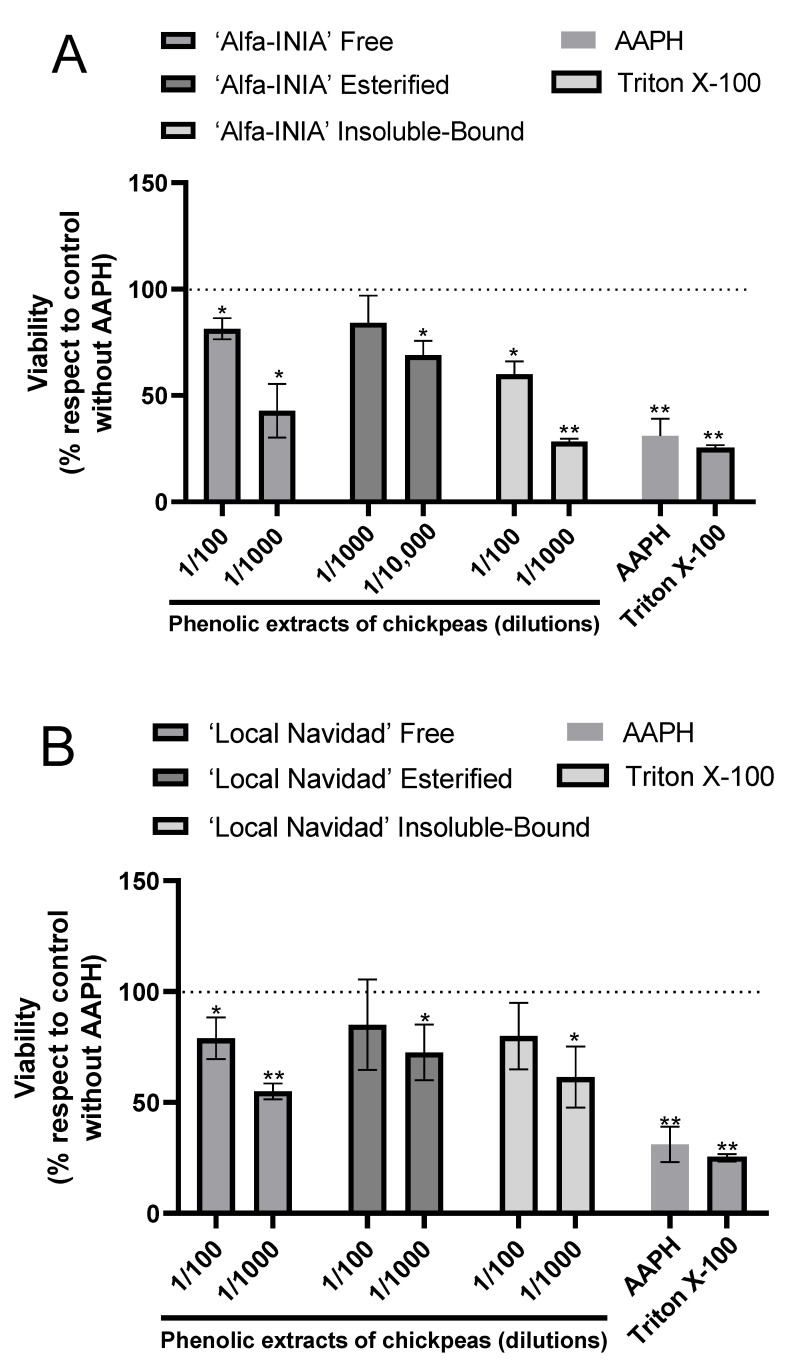

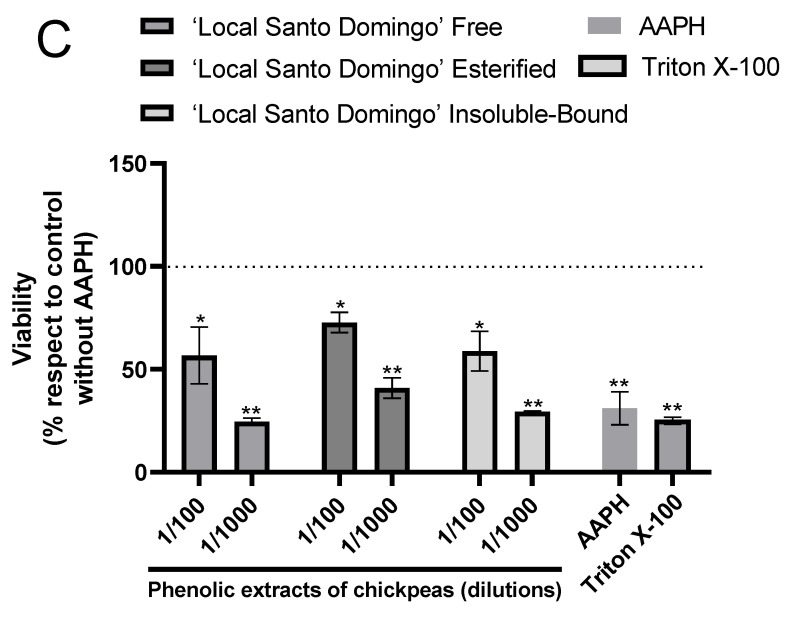
Phenolic extracts of chickpeas prevents AAPH-induced cell death. Cell viability evaluated by Alamar blue of HUH-7 cells treated with phenolic extracts of chickpeas of ‘Alfa-INIA’ (**A**), ‘Local Navidad’ (**B**) and ‘Local Santo Domingo’ (**C**) at different dilutions (1/100, 1/1000 or 1/10,000) and co-treatment with 2,2′-Azobis (2-amidinopropane) dihydrochloride (AAPH) for 24 h at 200 mM. Positive control of cell death, cells treated with Triton X-100 at 1% for 10 min. Data are expressed as percentage of viability with respect to the control cells (Cells without AAPH). Data are shown as mean ± SD (n = 3). A one-way ANOVA statistical test was performed followed by Tukey test. Statistically significant differences compared to the control group (cells without treatment) (* *p* < 0.05, ** *p* < 0.01).

According to hepatoprotective potential, the results showed the protective effects of phenolic extracts of chickpeas at two different concentrations against AAPH-induced free radical’s accumulation in HUH-7 cells (Figure 3).

As for ‘Alfa-INIA’ chickpeas, the free and insoluble-bound phenolic compounds at a dilution of 1/100 protected against cell death induced by AAPH. Likewise, esterified phenolics ‘Alfa-INIA’ at dilutions of 1/1000 and 1/10,000 prevented cell AAPH-induced cell death (Figure 3A). ‘Local Navidad’ chickpeas (free compounds at 1/100 dilution, esterified and insoluble-bound compound at two different dilutions (1/100 and 1/1000)) prevented cell death induced by AAPH (Figure 3B). Finally, free, esterified and insoluble-bound phenolic extracts from ‘Local Santo Domingo’ chickpeas at 1/100 dilution also prevented cell death induced by AAPH (Figure 3C).

## 4. Discussion

TPC allows the estimation of the content or presence of phenolic compounds in a sample. Phenolic compounds exhibit redox properties responsible for their antioxidant characteristics. In all the varieties tested, TPC values were found to be higher compared to those of other reported chickpeas varieties [15,16,34], but within the range of those obtained by Johnson et al. [35]. These differences may be due to the grain type being from distinct varieties, harvest conditions, and extraction methods. In addition, we observed distinctive differences in free, esterified, and IBP fractions. These metabolites have been identified in different legumes [11] and those of ‘Local Santo Domingo’ showed the highest TPC in this work.

In the FRAP method, the reduction from Fe^3+^ to Fe^2+^ is monitored for measuring the antioxidant capacity. Ferric ions are associated with the oxidation of proteins [36] and lipids [37]. The hydroxyl radicals and ferric ions are produced through the Fenton reaction in the presence of ferrous ions and hydrogen peroxide. The ability of phenolic compounds to chelate ferrous ions has been described in previous studies [38]. Some spectroscopic methods (Fourier-transform infrared spectroscopy and electrospray ionization mass spectrometry) and thermogravimetric analysis have been used to monitor the metal chelation properties of some isoflavonoids (genistein and biochanin) [39]. In this study, the phenolics present may have served as ferrous ion chelators; thus chickpeas phenolic compounds may reduce ferric ions to ferrous ions, altering their ratios and arresting or retarding the Fenton reaction. Products of a Fenton reaction (ferric ions and hydroxyl radicals) can cause DNA damage in addition to the oxidation of proteins and lipids [36,37,40]. Therefore, the phenolic compounds present in all chickpeas samples tested have potential biological activity.

In this study, the observed FRAP values were lower than other varieties reported in the literature [13,34]. However, it is difficult to establish a comparison between them by only analyzing the same fractions, due to the differences in varieties, geographical cultivation regions, and extraction methods. ‘Local Santo Domingo’ showed higher FRAP levels in comparison with other varieties and it corresponds with the level of TPC. Moreover, the FRAP values in the soluble and IBP fractions were different among all varieties and were higher in IBP in all varieties except the ‘Local Navidad’, although it had a high TPC content in this fraction.

It is very difficult to compare TPC and FRAP levels among different varieties, even within the four varieties in this work. Moreover, there are no evaluation studies on the three phenolic fractions for TPC and FRAP of Chilean chickpeas; thus, this may be considered as the first one on the topic. Therefore, we cannot compare them with other studies with the same field experimental conditions and varieties. Nonetheless, all differences, ‘Local Santo Domingo’, ‘Local Navidad’, ‘Alfa-INIA’, and ‘California-INIA’, showed satisfactory phenolic contents and antioxidant capacities.

The ORAC measurement is widely used in the presence of ROS, especially peroxyl radicals, which are important in food and biological systems [41]. All tested varieties in the three fractions were high in IBP fractions. ‘California-INIA’ and ‘Local Navidad’ were the varieties with the highest ORAC. Furthermore, our results were higher than those reported by Xu et al. [34] but within the range described by Heiras et al. [15]. All these differences were associated with the intrinsic characteristics of each variety.

Phenolic acids, flavonoids, and isoflavonoids were putatively detected using LC-MS/MS (Table 2). The MRM method scans specific compounds in SIM mode. Using authentic standards, we identified these metabolites according to their mass spectral characteristics. Meanwhile, two MRM transitions were considered and 15 compounds recognized by the molecular and other specific ions. The scanned m/z of compounds coincided with those previously reported in chickpeas [21] and other legumes [34].

Various phenolic compounds were detected using LC-MS/MS (Table 3). Phenolic acids are important compounds in legumes, cereals, and seeds. Studies have described the high content of phenolic acids in legumes, principally in the IBP fraction [14]. These metabolites have antioxidant and antimicrobial properties, including protection against biotic and abiotic factors. In all studied varieties, the most prominent phenolic acids were m-hydroxybenzoic acid and *p*-coumaric acid. They were present in the free, esterified, and IBP fractions. Meanwhile, m-hydroxybenzoic acid was the phenolic acid with the highest concentration. These acids were found in other chickpea seed extracts [42]. However, these authors did not address the presence of soluble esterified phenolic acids. Other phenolic acids, such as cinnamic and sinapic acids, were quantified only in the free fraction, while being absent in the esterified and IBP fractions. There are no studies of the phenolic acid profile in the four varieties tested in this study for the first time.

Flavonoids are essential phenolic compounds in plants. They participate in different functions such as growth, defense, physical, and aromatic characteristics [43]. In addition, they can modulate cell metabolism, functions associated with antioxidant and anti-inflammatory properties [44]. From the five flavonoids found, only taxifolin was quantifiable in the fractions of all varieties, except in esterified and IPB of ‘Alfa-INIA’. Regardless (free, esterified, or insoluble-bound), to the best of our knowledge, this is the first identification and quantification of taxifolin in chickpeas. Other flavonoids, such as luteolin, kaempferol, rutin, and isorhamnetin, were detected only in trace amounts in all chickpea varieties. In previous studies, kaempferol was present in ‘California-INIA’ and ‘Local Navidad’ in the esterified fractions. Nevertheless, we did not detect kaempferol in the free phenolic fraction of ‘California-INIA’ and ’Alfa-INIA’. In addition, rutin was found in the soluble fractions in the three evaluated varieties (‘California-INIA’, ’Alfa-INIA’, and ‘Local Navidad’) [21]. Insoluble-bound kaempferol, rutin, and isorhamnetin were detected in all varieties, but insoluble-bound luteolin was absent. Kaempferol is widely recognized for its modulation of inflammatory responses, angiogenesis, and apoptosis process [7]. It has been associated with the improvement in different conditions or diseases such as post-menopausal bone loss [45], obesity [46], diabetes [47], and cancer [48]. Rutin is another antioxidant compound with various functions at the system levels such as nervous, gastrointestinal, cardiovascular, respiratory, and immune [49], and is thus considered a potential pharmacological substance. According to Gong et al. [50], the mechanisms of action of isorhamnetin are explained by their anti-inflammation, and antioxidation properties.

Isoflavonoids are a group of flavonoids present in legume seeds and are associated with prevention of cancer and cardiovascular ailments [51]. In this study, we detected four isoflavonoids in all chickpea varieties. As far as we know, this is the first study reporting the presence of insoluble-bound isoflavones in chickpeas. From these compounds, biochanin A was present at the highest level and is a bioactive compound present in different legumes. It is associated with the cell cycle [52] and other molecular pathways linked to transcriptional factors (NF-kB and PPAR γ) [53,54]. This isoflavonoid has therapeutic potential and its activity has been studied in different models [55]. However, its clinical use has been limited due to its low bioavailability [56].

Phenolic compounds present in legume seeds have shown positive effects as inhibitors of DNA damage induced by peroxyl radicals [42,57]. Moreover, soluble phenolics from fermentative processes have not been demonstrated as antioxidants in oxidative DNA damage prevention [58]. These results are an important key in avoiding or preventing cancer initiation. The DNA damage signaling/repair pathways are associated with the etiology of human cancers [59]. DNA damage may affect the process of the replication and transcription of DNA and end in mutagenesis [60].

The presence of different phenolic acids, flavonoids, and isoflavonoids contributes to elevating the chickpea’s bioactive characteristics associated with antioxidant capacity. The Chilean landraces and INIA (Chile’s National Agricultural Research Institute) varieties of chickpeas show various phenolic compounds that present reducing power and scavenge of peroxyl radicals. To confirm these results in a biological model, HuH-7 cells were treated with free phenolics as well as those released from their esterified form. Regardless of the fraction and concentration, which ranged from 1/100 to 1/10,000, all extracts decreased the oxidative damage induced by AAPH (24.5–85.0%). In general, a concentration-dependent effect was observed with extracts at higher concentrations being most effective.

Besides the antiradical properties, phenolic extracts tested here may act by other mechanisms, which deserves further investigation. It has been reported that there is a relationship between the hepatoprotective effect of some phenolic compounds and their antioxidant capacity [61]. Hepatotoxicity can be induced by drugs/chemicals such as acetaminophen, isoniazid, thioacetamide, carbon tetrachloride (CCl_4_), and D-galactosamine [62,63] as well as by mycotoxins, especially aflatoxin B1, which may be found in contaminated food and feed. The liver is most susceptible to damage induced by aflatoxin B1 since its activation takes place in this organ [64].

Similar to other drugs and/or chemicals, mycotoxins induce oxidative stress. The antioxidant and hepatoprotective effect of biochanin A, the main phenolic present in chickpeas, was summarized by Raheja et al. [62]. Cinnamic acid and its derivatives also showed hepatoprotective effects in Wistar rats treated with CCl_4_. Phenolic antioxidants can inhibit the generation of free radicals, which is important in liver protection. Our study demonstrates that the free, esterified, and insoluble-bound of chickpea possess potent hepatoprotective effects against AAPH-induced cytotoxicity, probably due to their significant antioxidant activity. The protective effect of phenolics from chickpeas against other free radical generators is therefore encouraged. Likewise, aspects such as bioavailability and future in-vivo studies should also be considered.

## 5. Conclusions

A comprehensive characterization of soluble free, soluble esterified, and insoluble-bound phenolic acids, flavonoids, and isoflavonoids of four chickpea Kabuli-type varieties of chickpeas grown in Chile is reported for the first time. In general, the main fraction contributing to total phenolic content, reducing power, and antiradical activity was the one recovered from the insoluble-bound form. Furthermore, m-Hydroxybenzoic acid, cinnamic acid, *p*-coumaric acid, ferulic acid, syringic acid, sinapic acid, daidzein, formononetin, genistein, biochanin A, luteolin, kaempferol, taxifolin, isorhamnetin, or rutin were identified in at least one of the three studied fractions evaluated. From these, m-hydroxybenzoic acid, taxifolin, and biochanin A were the main phenolics found while taxifolin is reported for the first time in chickpeas. Biochanin A was the main phenolic in the free phenolic fraction while the fraction released from the insoluble-bound form contained mainly m-hydroxybenzoic acid. Lending support to the results found in the antiradical activity towards peroxyl radicals, all fractions showed hepatoprotection in HuH-7 cells exposed to a generator of this reactive oxygen species.

## Figures and Tables

**Table 1:** Parameters used for the LC–MS/MS analysis of the phenolics examined.

Compound	MRM Transition 1	DP *	CE	CXP	MRM Transition 2	DP	CE	CXP
*m*-Hydroxybenzoic acid	137.0 > 92.9	−50	−16	−7	137.0 > 64.9	−50	−32	−11
Cinnamic acid	147.0 > 103.1	−55	−14	−7	147.0 > 76.9	−55	−28	−7
*p*-Coumaric acid	162.9 > 119	−70	−20	−5	162.9 > 119	−70	−38	−25
Ferulic acid	193.0 > 134.0	−55	−20	−7	193.0 > 177.9	−55	−16	−15
Syringic acid	197.0 > 181.9	−65	−18	−5	197.0 > 122.9	−65	−30	−7
Sinapic acid	223.0 > 207.9	−75	−18	−7	223.0 > 148.8	−75	−26	−13
Daidzein	252.9 > 131.7	−105	−50	−9	252.9 > 207.7	−105	−44	−1
Formononetin	267.1 > 251.6	−110	−26	−9	267.1 > 222.9	−110	−46	−9
Genistein	268.8 > 133.0	−170	−38	−43	268.8 > 181.0	−170	−34	−13
Biochanin A	282.9 > 267.9	−80	−32	−5	282.9 > 211.1	−80	−46	−5
Luteolin	285.0 > 133.0	−125	−42	−5	285.0 > 150.9	−125	−34	−11
Kaempferol	285.0 > 184.9	−135	−36	−15	285.0 > 116.9	−135	−48	−3
Taxifolin	302.9 > 285.0	−105	−14	−5	302.9 > 125.0	−105	−30	−7
Isorhamnetin	315.0 > 299.9	−130	−32	−15	315.0 > 150.9	−130	−40	−11
Rutin	609.0 > 299.8	−170	−50	−13	609.0 > 300.5	−170	−42	−9

* DP, declustering potential; CE, collision energy; CXP, collision cell exit potential; MRM, multiple reaction monitoring.

**Table 2:** The TPC (mg GAE/100 g), FRAP (μmol TE/100 g) and ORAC (μmol TE/100 g) of soluble (free and esterified) and insoluble-bound compounds in chickpeas.

	Free	Esterified	Insoluble-Bound	Total
TPC				
‘California-INIA’	7.3 ± 0.2 b	4.5 ± 0.3 c	13.3 ± 0.3 a	25.1
‘Alfa-INIA’	8.9 ± 0.7 a	1.6 ± 0.1 c	6.8 ± 0.4 b	17.3
‘Local Navidad’	7.7 ± 0.4 b	3.0 ± 0.3 c	12.2 ± 1.0 a	22.9
‘Local Santo Domingo’	10.2 ± 0.7 b	4.6 ± 0.2 c	16.7 ± 0.4 a	31.5
FRAP				
‘California-INIA’	14.6 ± 0.4 b	9.9 ± 0.4 c	19.6 ± 0.7 a	44.1
‘Alfa-INIA’	16.3 ± 0.9 b	7.7 ± 0.3 c	28.7 ± 0.8 a	52.7
‘Local Navidad’	16.2 ± 0.6 a	9.0 ± 0.6 b	8.3 ± 0.2 b	33.4
‘Local Santo Domingo’	19.4 ± 0.3 b	9.1 ± 0.4 c	41.8 ± 0.4 a	70.4
ORAC				
‘California-INIA’	181.8 ± 12.1 b	69.0 ± 2.9 c	1798.4 ± 38.1 a	2049.3
‘Alfa-INIA’	221.4 ± 9.9 b	23.5 ± 0.7 c	461.6 ± 57.2 a	706.5
‘Local Navidad’	257.0 ±13.8 b	60.7 ± 6.4 c	1243.1 ± 34.9 a	1560.8
‘Local Santo Domingo’	328.2 ± 21.9 b	103.5 ± 4.6 c	391.6 ± 1.9 a	823.2

Data are presented as mean ± SD (n = 3). Values within the same column part followed by the same capital letters are not significantly different (*p* < 0.05). Values within the same row followed by the same small letters are not significantly different (*p* < 0.05). Abbreviation: TPC, total phenolic content; FRAP, ferric reducing antioxidant power; ORAC, oxygen radical absorbance capacity.

**Table 3:** Phenolic acids, flavonoids, and isoflavonoids soluble (free and esterified) and insoluble-bound compounds in chickpeas (µg/100 g).

	Free	Esterified	Insoluble-Bound	Total
**Phenolic acids**				
m-Hydroxybenzoic acid **				
‘California-INIA’	81.4 ± 2.0 b	67.0 ± 2.0 b	309.7 ± 11.6 a	458.0
‘Alfa-INIA’	66.0 ± 2.8 b	38.4 ± 4.0 b	294.2 ± 27.1 a	398.6
‘Local Navidad’	64.4 ± 0.9 b	59.3 ± 1.4 b	319.1 ± 29.5 a	442.8
‘Local Santo Domingo’	23.3 ± 2.4 c	50.7 ± 3.9 b	143.2 ± 3.5 a	217.2
Cinnamic acid **				
‘California-INIA’	2.2 ± 0.2	nd	nd	2.2
‘Alfa-INIA’	2.8 ± 0.1	nd	nd	2.8
‘Local Navidad’	1.6 ± 0.1	nd	nd	1.6
‘Local Santo Domingo’	tr	nd	nd	tr
*p-Coumaric acid*				
‘California-INIA’	16.3 ± 0.4 a	8.7 ± 0.8 b	17.3 ± 1.9 a	42.3
‘Alfa-INIA’	8.6 ± 0.6 a	0.6 ± 0.1 c	6.0 ± 0.1 b	15.2
‘Local Navidad’	9.6 ± 0.5 a	1.7 ± 0.1 c	4.1 ± 0.1 b	15.4
‘Local Santo Domingo’	22.7 ± 0.4 a	10.4 ± 1.0 c	6.6 ± 0.4 b	39.7
*Ferulic acid*				
‘California-INIA’	5.8 ± 0.1 a	2.7 ± 0.2 b	5.4 ± 0.7 a	13.8
‘Alfa-INIA’	3.4 ± 0.2 a	0.9 ± 0.0 b	tr	4.3
‘Local Navidad’	2.1 ± 0.1 a	0.6 ± 0.0 b	tr	2.7
‘Local Santo Domingo’	nd	2.3 ± 0.2	tr	2.3
*Syringic acid*				
‘California-INIA’	1.2 ± 0.0 c	4.0 ± 0.1 b	7.5 ± 0.8 a	12.8
‘Alfa-INIA’	1.2 ± 0.0 b	1.3 ± 0.2 b	2.3 ± 0.1 a	4.8
‘Local Navidad’	1.4 ± 0.1 c	4.0 ± 0.2 b	5.8 ± 0.6 a	11.2
‘Local Santo Domingo’	0.6 ± 0.0 c	4.6 ± 0.2 a	2.1 ± 0.0 b	7.3
*Sinapic acid*				
‘California-INIA’	12.9 ± 0.7	nd	nd	12.9
‘Alfa-INIA’	49.4 ± 0.6	nd	nd	49.4
‘Local Navidad’	33.3 ± 2.0	nd	nd	33.3
‘Local Santo Domingo’	23.6 ± 0.8	nd	nd	23.6
**Flavonoids**				
*Luteolin*				
‘California-INIA’	tr	tr	nd	tr
‘Alfa-INIA’	tr	nd	nd	tr
‘Local Navidad’	tr	nd	nd	tr
‘Local Santo Domingo’	tr	tr	nd	tr
*Kaempferol*				
‘California-INIA’	tr	tr	tr	tr
‘Alfa-INIA’	tr	tr	tr	tr
‘Local Navidad’	tr	tr	tr	tr
‘Local Santo Domingo’	tr	tr	tr	tr
Taxifolin **				
‘California-INIA’	5.3 ± 0.3 c	14.5 ± 1.4 b	22.9 ± 2.1 a	42.6
‘Alfa-INIA’	9.4 ± 0.5	nd	nd	9.4
‘Local Navidad’	4.6 ± 0.1 b	3.5 ± 0.1 b	45.5 ± 1.2 a	53.6
‘Local Santo Domingo’	89.0 ± 2.0 a	9.9 ± 0.4 c	56.6 ± 3.9 b	155.5
*Isorhamnetin*				
‘California-INIA’	tr	tr	tr	tr
‘Alfa-INIA’	tr	tr	tr	tr
‘Local Navidad’	tr	tr	tr	tr
‘Local Santo Domingo’	tr	tr	tr	tr
*Rutin*				
‘California-INIA’	tr	tr	tr	tr
‘Alfa-INIA’	tr	tr	tr	tr
‘Local Navidad’	tr	tr	tr	tr
‘Local Santo Domingo’	tr	tr	tr	tr
**Isoflavonoids**				
*Daidzein*				
‘California-INIA’	tr	nd	nd	tr
‘Alfa-INIA’	tr	nd	nd	tr
‘Local Navidad’	tr	nd	nd	tr
‘Local Santo Domingo’	tr	nd	nd	tr
Formononetin **				
‘California-INIA’	140.8 ± 9.0	nd	tr	140.8
‘Alfa-INIA’	59.6 ± 3.8	nd	tr	59.6
‘Local Navidad’	128.6 ± 7.0	nd	tr	128.6
‘Local Santo Domingo’	51.9 ± 2.9	nd	tr	51.9
*Genistein*				
‘California-INIA’	nd	tr	tr	tr
‘Alfa-INIA’	nd	tr	tr	tr
‘Local Navidad’	nd	tr	tr	tr
‘Local Santo Domingo’	nd	tr	tr	tr
Biochanin A **				
‘California-INIA’	7586.4 ± 632.3 a	286.5 ± 13.0 b	507.1 ± 57.7 b	8380.0
‘Alfa-INIA’	5401.1 ± 418.4 a	139.1 ± 5.2 c	841.9 ± 85.1 b	6382.1
‘Local Navidad’	6048.0 ± 290.4 a	130.2 ± 6.8 b	117.9 ± 17.1 b	6296.1
‘Local Santo Domingo’	7544.0 ± 15.2 a	233.1 ± 8.7 c	583.7 ± 14.5 b	8360.8

* tr, trace; nd, not detected. ** m-Hydroxybenzoic acid, taxifolin, biochanin A, and formononetin were quantified as equivalents of gallic acid, quercetin, genistein, and daidzein, respectively.

## Data Availability

The data presented in this study are available on request from the first author and J.E.O.
